# Significance of Beta-Band Oscillations in Autism Spectrum Disorders During Motor Response Inhibition Tasks: A MEG Study

**DOI:** 10.1007/s10548-020-00765-6

**Published:** 2020-04-17

**Authors:** Vera Moliadze, Alla Brodski-Guerniero, Magdalena Schuetz, Julia Siemann, Ekaterina Lyzhko, Sabine Schlitt, Janina Kitzerow, Anne Langer, Jochen Kaiser, Marcus J. Naumer, Michael Wibral, Jason Chan, Christine M. Freitag, Michael Siniatchkin

**Affiliations:** 1 Institute of Medical Psychology and Medical Sociology, University Medical Center Schleswig Holstein, Kiel University, Kiel, Germany; 2 Department of Child and Adolescent Psychiatry, Psychosomatics and Psychotherapy, Autism Research and Intervention Center of Excellence, University Hospital Frankfurt, Goethe University, Frankfurt am Main, Germany; 3 MEG Unit, Brain Imaging Center, University Hospital Frankfurt, Goethe University, Frankfurt am Main, Germany; 4 Department of Child and Adolescent Psychiatry and Psychotherapy, Ev. Hospital Bethel, Bielefeld, Germany; 5 Institute of Mathematical Problems of Biology RAS - the Branch of Keldysh Institute of Applied Mathematics of Russian Academy of Sciences, Pushchino, Moscow Region, Russia; 6 Institute of Medical Psychology, Faculty of Medicine, University Hospital Frankfurt, Goethe University, Frankfurt am Main, Germany; 7 University of Koblenz-Landau, Landau (Pfalz), Germany; 8 Campus Institute for Dynamics of Biological Networks, Georg-August University, Goettingen, Germany; 9 School of Applied Psychology, University College Cork, Cork, Ireland

**Keywords:** ASD, MEG, Beta-band oscillations, Motor response inhibition

## Abstract

In Autism Spectrum Disorders (ASD), impaired response inhibition and lack of adaptation are hypothesized to underlie core ASD symptoms, such as social communication and repetitive, stereotyped behavior. Thus, the aim of the present study was to compare neural correlates of inhibition, post-error adaptation, and reaction time variability in ASD and neuro-typical control (NTC) participants by investigating possible differences in error-related changes of oscillatory MEG activity. Twelve male NTC (mean age 20.3 ± 3.7) and fourteen male patients with ASD (mean age 17.8 ± 2.9) were included in the analysis. Subjects with ASD showed increased error-related reaction time variability. MEG analysis revealed decreased beta power in the ASD group in comparison to the NTC group over the centro-parietal channels in both, the pre-stimulus and post-response interval. In the ASD group, mean centro-parietal beta power negatively correlated with dimensional autism symptoms. In both groups, false alarms were followed by an early increase in temporo-frontal theta to alpha power; and by a later decrease in alpha to beta power at central and posterior sensors. Single trial correlations were additionally studied in the ASD group, who showed a positive correlation of pre-stimulus beta power with post-response theta, alpha, and beta power, particularly after hit trials. On a broader scale, the results deliver important insights into top-down control deficits that may relate to core symptoms observed in ASD.

## Introduction

In Autism Spectrum Disorder (ASD) abnormal cognitive processing of several tasks has been described, including impaired response inhibition, post-error adaptation, and increased performance variability. The present study aims at eliciting the underlying neural mechanisms using source reconstruction and time–frequency analysis of MEG data.

Response inhibition describes the termination of an automated or pre-potent response elicited by an external stimulus. This function is, among others, required for the regulation of affect and the conscious inhibition of impulses in a social context. Deficient response inhibition has been discussed to underlie ASD related repetitive and stereotyped behavior, including motor stereotypies (Chmielewski and Beste 2015; LeMonda et al. 2012; Schmitt et al. 2018). In addition social communication deficits inherent in ASD may also be related to abnormal response inhibition, mediated by the failure to suppress inappropriate social reactions (Chmielewski and Beste 2015; Vara et al. 2014), especially in children and adolescents (Sachse et al. 2013; Weiss 2006).

In neuro-typical control (NTC), electroencephalographic (EEG) and functional magnetic resonance imaging (fMRI) based brain coherence measures have been established as neural correlates of inhibitory control (fMRI: Bogler et al. 2017; Mennes et al. 2011; EEG: Gonzalez-Castillo and Bandettini 2017; Klimesch 1999). Strength of alpha coherence correlated with decision-making and strength of theta coherence correlated with response inhibition abilities (Harmony et al. 2009; Shibata et al. 1998). In ASD neural connectivity during response inhibition has rarely been studied (Kana et al. 2014). Studies on task-related connectivity have found reduced connectivity strength of low frequency bands (Doesburg et al. 2013; Murias et al. 2007) and increased phase synchrony at high frequencies (Buard et al. 2013). With respect to brain-behavior relations, elevated theta coherence in ASD correlated with error rates (Han and Chan 2017), and increased functional connectivity was associated with a reactive control mode in ASD (Solomon et al. 2017). Reduced connectivity of low frequency bands points to aberrant long-range connectivity in ASD (Khan et al. 2013; Vissers et al. 2012). Still, other studies found a contrasting pattern, with enhanced connectivity at low (Chan et al. 2011) and reduced synchrony at high frequencies (Peiker et al. 2015).

In addition to impaired response inhibition, ASD is associated with abnormal adaptive behaviors after unsuccessful inhibition (i.e., errors). Typically, post-error slowing represents a regulatory mechanism triggered by an attentional shift preceding errors in order to enhance top down behavioral control in healthy subjects (Amengual et al. 2013). Post-error slowing corresponds with the EEG or MEG based evoked potential “Error-Related Negativity” (ERN, Nieuwenhuis et al. 2001), which may reflect enhanced frontal theta power (Cavanagh et al. 2010). In ASD, impairments in post-error slowing (Sokhadze et al. 2010) and a reduced and delayed EEG-based ERN (Vlamings et al. 2008); were observed. To date no study has explicitly studied connectivity patterns during post-error adaption in ASD.

Likewise, there is a gap in the literature with respect to performance instability in ASD, which yielded controversial discussions due to mixed study results. Thus, enhanced variability was found in some studies (Christakou et al. 2013; Dinstein et al. 2012), contrasting with evidence for unaltered variability reported elsewhere (Geurts and Vissers 2012; Lundervold et al. 2016) Diverse neurophysiological parameters also appear to be more variable in ASD, including EEG fluctuations and BOLD signal-to-noise ratio (see review by Karalunas et al. (2014). However, there is little evidence so far about specific effects of these altered brain arousal states on performance measures in ASD despite recent suggestions of variability being an endophenotype of ASD (David et al. 2016). In particular, few studies explicitly related intra-individual performance with EEG variability (Lushchekina et al. 2016; Papenberg et al. 2013).

The aim of the current study was to study ASD related neural signatures based on MEG oscillatory dynamics during a Go-NoGo task, which allows assessing response inhibition, post-error adaptation, and response variability. Error-related changes of oscillatory MEG activity were compared between NTC and ASD patients, focusing on oscillatory activity in the theta, alpha and beta frequency range in ASD. Previously, alpha and theta bands have been suggested to contribute to various aspects of attention, orienting, and cognitive control in typically developing children and adults (Klimesch et al. 2007; Mazaheri et al. 2009). In addition, attenuation of beta activity over contralateral sensorimotor areas seems to precede voluntary movement and motor preparation (e.g. Kilavik et al. 2013; Tzagarakis et al. 2015).

We tested the following specific hypotheses. We expected to observe increased performance variability (hypothesis 1), reduced post-error adaptation (hypothesis 2), and reduced activity in attention-related frequency bands (hypothesis 3) in ASD. Additionally, we expected a positive correlation between pre-stimulus brain activity and post-response measures in both, ASD and NTC (hypothesis 4).

## Materials and methods

The study was approved by the Ethics Committee of the Faculty of Medicine of the Goethe University, Frankfurt am Main, Germany. All participants and their parents gave written informed consent in accordance with the Declaration of Helsinki on biomedical research involving human subjects (Tokyo amendment), and received monetary compensation for participation.

### Subjects

In total, 51 male participants were recruited for this study. The sample consisted of 21 patients (ASD) and 30 healthy NTC subjects. Three control subjects had to be excluded retrospectively because they were screened positive for psychiatric disorders. Another participant from the NTC group was excluded because he was unable to follow the task instructions. Additionally, after artifact rejection (excessive movement during measurements; extreme amount of muscle artifacts), 7 patients and 14 control subjects were excluded from analyses based on a low number of remaining trials. The final samples with which the analyses were performed are described below.

#### Patient Group (ASD)

Patients were recruited through the Department of Child and Adolescent Psychiatry, Psychosomatics, and Psychotherapy, University Hospital Frankfurt, Goethe University, Frankfurt am Main, Germany. Additionally, we found patients through online forums. The ASD group consisted of 14 subjects with a mean age of 17.8 years (± 2.9; range: 14.4–23.9 years) and a mean IQ of 109.24 (± 15.32; range: 87–145). Nine were right handed, one left handed, and four reported mixed handedness.

#### Control Group (NTC)

Healthy participants were recruited from local schools. In addition, the study was also advertised on the campus of the Goethe-University Frankfurt. The mean age of the 12 subjects in the NTC group was 20.3 years (± 3.7; range: 14.9–26.9). Their mean IQ was 112.86 (± 17.54; range: 88–135). Nine of them were right handed, one left handed, and two indicated having mixed handedness (see Table 1 for subjects’ characteristics).

**Table 1:** Summary of group characteristics

	ASD	NTC	Statistics
Age	17.8 ± 2.9	20.3 ± 3.7	p = 0.07
IQ	109.4 ± 16.7	113.9 ± 19.7	p = 0.53
EQ	22.3 ± 11.5	43.4 ± 11.4	p = 0.0013*
AQ	17.4 ± 3.3	4.8 ± 7.07	p = 0.00009*

*EQ* empathy quotient (Baron-Cohen and Wheelwright 2004), *AQ* autism spectrum quotient (Baron-Cohen et al. 2001)

*Bold values indicate significant results of Wilcoxon ranked sum test

### Diagnostic Tests and Monitoring Tools

To capture background variables, to ensure comparability of the ASD and NTC group, and to confirm mental health (NTC) and correct diagnosis (ASD), several questionnaires and observational methods were used (see below). Handedness was assessed by the Edinburgh Handedness Inventory (Oldfield 1971). Additionally, subjects answered questions pertaining to their health (including questions about medication or drug use) and their socioeconomic background.

#### ASD Group

ASD patients were diagnosed by experienced clinicians according to ICD-10 (World Health Organization 1992) based on the German version of the Autism Diagnostic Observation Schedule (ADOS, Rühl et al. 2004) and/or the Autism Diagnostic Interview-Revised (ADI-R, Bölte et al. 2006).

For ASD patients not previously diagnosed at Frankfurt, parents were first asked to fill out the German versions of the Social Communication Questionnaire (SCQ; Bölte 2006; Rutter et al. 2003) and the Social Responsiveness Scale (SRS; Bölte et al. 2005; Constantino and Gruber 2002). If these screening instruments were positive, ADOS module 3 or 4 was performed by a trained clinician to confirm the current ASD diagnosis.

Severity of current autistic traits was studied by self-report: The short German version of the Autism Spectrum Quotient (AQ) was implemented consisting of 33 Items, which are summarized to one single score. Retest reliability (r_t_ = 0.79) and concurrent validity are established (Freitag et al. 2007). In addition the empathy quotient (EQ; Baron-Cohen and Wheelwright 2004) was applied (translated to German by Christine M. Freitag und K. Leistenschneider https://docplayer.org/48877515-Adult-asperger-assessment-deutsch-aaa-d.html).

#### NTC Group

A general screening questionnaire was used to ensure the mental health of our NCT group. The younger participants received the German version of the Youth Self Report (YSR; Arbeitsgruppe Deutsche Child Behavior Checklist 1998a; original version: Achenbach and Edelbrock 1991) and participants of 18 years or older received the German version of the Young Adult Self Report (YASR 18–30; Arbeitsgruppe Deutsche Child Behavior Checklist 1998b; original version: Achenbach 1997). Both self-report measures (Achenbach and Edelbrock 1991) cover nine syndrome scales (Withdrawal, Aggressive Behavior, Anxious/Depressed Symptoms, Somatic Complaints, Delinquent Behavior, Social Problems, Self-destructive/Identity Problems, Thought Problems, and Attention Problems) yielding two second order scales (Internalizing and Externalizing Symptoms). Subjects were excluded when they met clinical symptom criteria on at least one syndrome scale or one second order scale.

### Stimuli and Stimulus Presentation

To establish comparability with a previous study, the applied paradigm was analogous to that of Mazaheri et al. (2009).

#### Stimulus Parameters

Single white digits between 1 and 9 were presented on a black background. Each stimulus was displayed for 0.2 s with an average inter-stimulus interval of 1.5 s (randomly jittered between 1.3 and 1.7 s), during which a white fixation cross was displayed. The visual stimuli were projected onto a translucent screen using an LCD projector (Epson-EB-G5100, EPSON Deutschland GmbH, Meerbusch, Germany) with a refresh rate of 60 Hz. For this purpose, the projector was located outside the MEG chamber and directed the images inside the chamber onto the screen using two front-silvered mirrors. The screen was mounted at a viewing distance of 53 cm in front of the participant and stimuli subtended 4° of visual angle. Stimuli were controlled via the Presentation software package (Neurobehavioral Systems, Version 14).

#### Task and Instructions

**Fig. 1:**
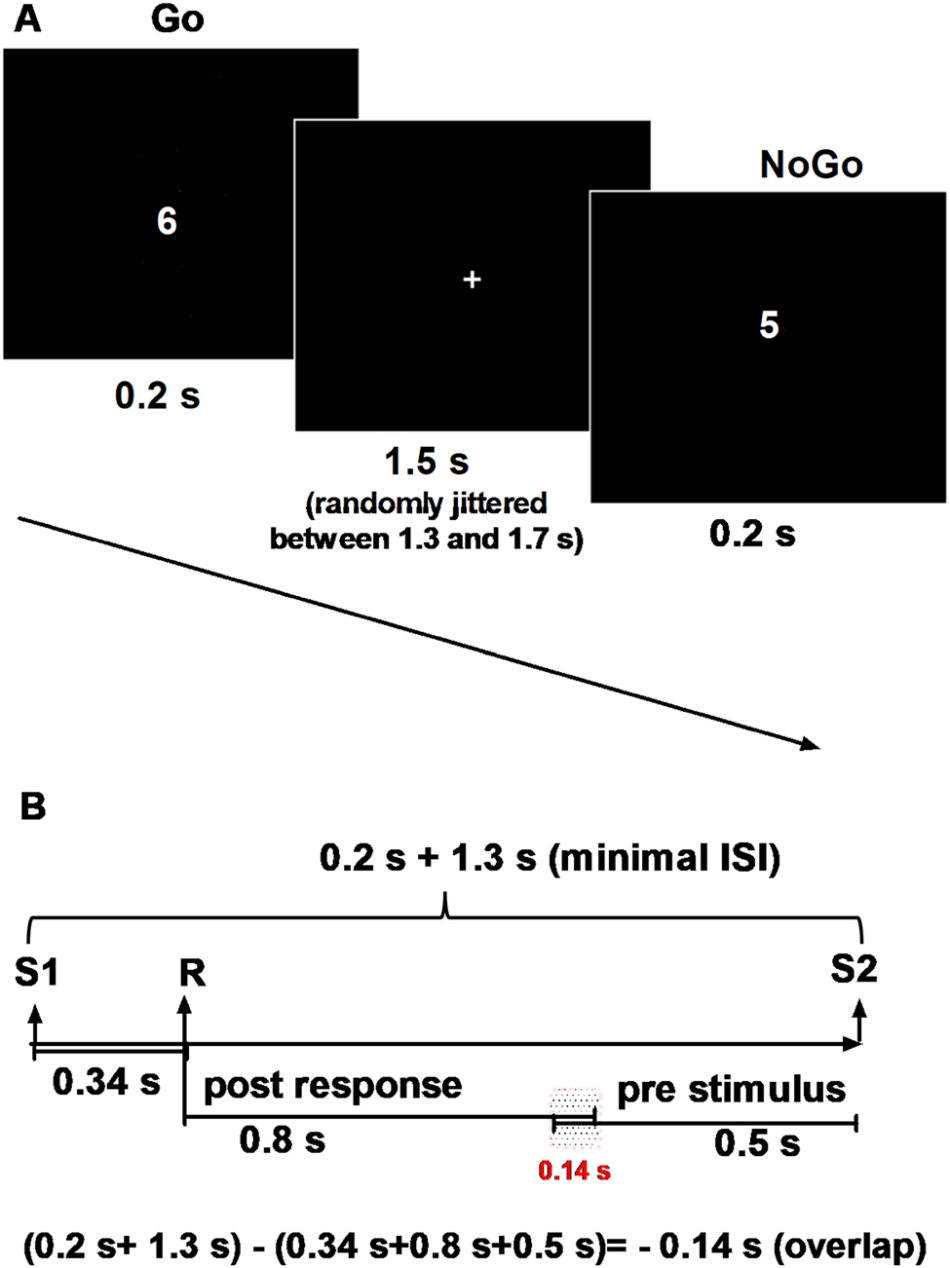
**a** Go/NoGo task: subjects were asked to respond to stimuli as quickly as possible by pressing a button as soon as a digit between 1 to 4 or 6 to 9 appeared (“Go” stimuli) and were told to withhold a button press when a “5” appeared (“NoGo” stimuli). Participants were instructed to keep their eyes focused on the fixation cross and to avoid any movement during the acquisition. Each stimulus was displayed for 0.2 s with an average interstimulus interval (ISI) of 1.5 s (randomly jittered between 1.3 and 1.7 s). **b** Time–frequency transformation was applied using adaptive sliding time-windows of 3 cycles per frequency between the time-interval of − 0.5 to 0.8 s around stimulus (stimulus interval) and response onset (response interval), respectively. A graphical depiction shows example in case reaction time 0.34 s and for shortest interstimulus interval 1.3. In this case we have overlap 0.14 s. For average interstimulus interval it will be no overlapping ((1.5 s + 0.2 s) − (0.34 s-0.8 s + 0.5 s) = 0.06 s).* S* stimulus, *R* response

Subjects performed a Go-NoGo task and responded by button press. They were asked to respond to stimuli as quickly as possible by pressing a button as soon as a digit between 1 to 4 or 6 to 9 appeared (“Go”-condition). They were told to withhold the button press when a 5 appeared (“NoGo”-condition). Single white digits between 1 and 9 were presented on a black background. Each stimulus was displayed for 0.2 s with an average inter-stimulus interval of 1.5 s (randomly jittered between 1.3 and 1.7 s), during which a white fixation cross was displayed (see Fig. 1a).

Participants were instructed to keep their eyes focused on the fixation cross and to avoid any movement during the acquisition. Overall, 800 trials were recorded in two blocks of 400 trials each, 160 (20%) of which were NoGo trials. The term Hits will subsequently be used to refer to button-presses during a Go-trial, while the term False Alarm (FA) will refer to commission errors, i.e. button-presses during a NoGo-trial. The term Correct Withholds (CW) will be used to describe correctly withholding button press in NoGo-trials.

### Data Acquisition

The acquisition of the MEG data was performed in line with the guidelines for MEG recordings (Gross et al. 2013). A whole-head system (Omega 2005; VSM MedTech, Port Coquitlam, BC, Canada) with 275 axial gradiometers was used to record MEG data. Signals were recorded continuously at a sampling rate of 1200 Hz in a synthetic third-order gradiometer configuration (Data Acquisition Software Version 5.4.0, VSM MedTech, BC, Canada). During the complete recording subjects’ head position relative to the gradiometer array was localized via three localization coils that were placed on the nasion and 1 cm anterior of the tragus of each ear. In order to detect artefacts the horizontal and vertical electrooculogram (EOG) and the electrocardiogram (ECG) were recorded via six electrodes. They were placed distal to the outer canthi of both eyes to record horizontal eye movements, above and below the right eye to record blinks and vertical eye movements, and below both collarbones to record the ECG. Impedances were kept below 15 kΩ as measured with an electrode impedance meter (Astro-Med Electrode Impedance Meter, Model F-EZM5, Grass Technologies, Natus Neurology Inc., Warwick RI, USA). Behavioral responses were recorded using a fiber optic response pad (Lumitouch, Photon Control, Burnbary, BC, Canada; later replaced with 8-Button Bimanual Fiber Optic Response Pad, Current Designs, Philadelphia, PA, USA), which was connected to the computer controlling stimulus presentation.

To obtain individual source grids for the source analysis, structural MR images were obtained with a 3 T Siemens Allegra or Trio scanner (Siemens Medical Solutions) using a standard T1 sequence (3D MPRAGE sequence, 176 slices, 1 × 1 × 1 mm voxel size). For the structural scans, vitamin E pills were placed at the former positions of the MEG localization coils to enable co-registration of MEG data and MR images.

### MEG Data Preprocessing and Analysis

Data analysis was performed with MATLAB (MATLAB 2008; The MathWorks) and the open source MATLAB toolbox FieldTrip (Oostenveld et al. 2011; version 2013 11-11). Data epochs were defined from − 1 s before the stimulus to 1.3 s after stimulus onset for the stimulus interval (CW; FA) and from − 1 s to 1.3 s around a button press for the response interval (Hits; FA). The projector delay of 0.045 s was taken into account in the analyses.

FieldTrip artifact-rejection routines were used to reject trials containing muscle or sensor jump artifacts automatically. To control the accuracy of the automatic artifact correction and to remove potential remaining artifacts, an additional visual artifact rejection was performed. After artifact rejection the mean head position over both experimental blocks was calculated for each subject and only trials in which the head position did not deviate more than 5 mm from the mean head position were considered for further analysis. This rather conservative procedure was supposed to reduce movement related inaccuracies. After trial rejection due to artifacts or subjects’ movement, the minimum amount of trials across the different conditions (CW and FA in the stimulus interval, Hits and FA in the response interval) was selected randomly from the available trials in each block (stratification). This procedure ensured that differences in the statistical analysis were not purely reflecting differential trial numbers. This procedure resulted in a mean trial number of 31 (± 18 SD) per condition. Additionally, to eliminate eye blink and eye movement related artifacts, an independent component analysis (ICA; Bell and Sejnowsk 1995; Makeig et al. 1996) was performed using the extended infomax (runica) algorithm implemented in fieldtrip/EEGLAB. ICA components strongly correlated with EOG and ECG channels were removed from the data. Finally, data was visually inspected for residual artefacts. To make our results comparable to those of (Mazaheri et al. 2009), we computed the planar representation of the data for the sensor level analysis. The horizontal and vertical components of the planar gradients were estimated at each sensor location by comparing the fields at the sensor and its neighboring sensors. Subsequently, the amplitude of each planar gradient was calculated by combining the orthogonal gradients (i.e. horizontal and vertical components) according to Pythagoras’ rule.

#### Time Frequency Analysis

For the time–frequency analysis, we used a sliding taper approach (Percival and Walden 1993) with Hanning tapers. Time frequency transformation was applied using adaptive sliding time-windows of 3 cycles per frequency in the time-interval of − 0.5 s to 0.8 s around stimulus (stimulus interval) and response onset (response interval), respectively. The time frequency representation was computed in 0.05 s time steps and 1 Hz frequency steps in the range between 3 and 30 Hz. No baseline correction was performed, as baseline (.i.e. pre-stimulus) activity was of interest for our hypotheses.

#### Source Analysis

Beamformer source analysis was performed using a frequency domain beamformer (Dynamic Imaging of Coherent Sources, DICS, Gross et al. 2001) implemented in the Fieldtrip toolbox. DICS analysis uses an adaptive spatial filter to estimate the power at every specific brain location. Before calculation of the DICS filter, an individual source grid was created for each subject by transforming the anatomical MR image to a standard T1 MNI template from the SPM8 toolbox (https://www.fil.ion.ucl.ac.uk/spm). The inverse of the resulting individual transformation matrix was then warped with a regular 3-D grid based on the T1 template (spacing 1 cm), resulting in an individual dipole grid for each subject in subject space. At all grid locations, lead fields were computed for each subject using a realistic single shell forward model (Nolte 2003). The spatial filter at each grid location was constructed from the individual lead fields and the cross-spectral density matrix for each subject (Gross et al. 2001). Beamformer filters were computed as “common filters” based on the data from all conditions. Spatial filtering of the sensor data was then performed by projecting data for each condition separately through the common filter for each condition. For source analysis, data from the axial sensors and not the planar gradients were used.

### Statistical Analysis

#### Statistical Analysis of Behavioral Data

To examine performance on a behavioral level, we investigated reaction times (RTs), variability of RTs (standard deviation of RTs), and error/correct response rates. For the estimation of main effects and interactions, a 1-within-1-between permutation Analysis of Variance (ANOVA; Suckling and Bullmore 2004), see (Brodski et al. 2015) for a recent application of permutation tests on behavioral and MEG data, was carried out with the within-subjects factor CONDITION (Hits vs FA) and between-subjects factor GROUP (NTC vs ASD). Non-parametric permutation tests are not based on the assumption of normality and are therefore recommended when testing behavioral data, which often do not follow a Gaussian distribution. In case of significant main effects of GROUP, CONDITION or the interaction of GROUP and CONDITION, post-hoc permutation t-tests were performed. The significance level was kept at p < 0.05. The number of permutations was set to 5000 for the ANOVA as well as the post-hoc t-tests.

#### MEG Sensor Level Statistics on Time Frequency Representations

Statistical analysis was performed on time–frequency representations using a 1within-1-between permutation ANOVA (Suckling and Bullmore 2004). The between-subjects factor was GROUP (NTC vs ASD) and the within-subjects factor was CONDITION, with the levels Correct withhold vs FA for the stimulus interval and Hits vs FA for the response interval, respectively. Before calculation of statistics, planar gradient power estimates for each subject and both conditions of interest were converted into z-values. Similar to the approach by Mazaheri et al. (2009) this aimed at normalizing the power values and thereby account for inter-individual variability in power.

For the stimulus interval the time from − 0.5 s before stimulus onset to stimulus onset was taken into account for statistical analysis at the sensor level, while for the response interval the time between the response and 0.8 s after the response was taken into account. For both time intervals, the frequency range from 3 to 30 Hz was considered for statistical analysis. A cluster-based correction method (Maris and Oostenveld 2007a) was used to account for multiple comparisons across channels, frequencies, and time. The parametric threshold for clustering was set to 0.01. The minimal number of neighbour channels in a cluster was set to 2. The sum of t-values in the clusters were tested against 1000 permuted datasets with a p-value below 0.05 (i.e. when > 95% of the permuted datasets did not show clusters with larger sums of t-values).

#### MEG Source Level Statistics

Sources were estimated for time–frequency ranges of interest based on the significant effects of a 1-within-1-between permutation ANOVA at the sensor level. Before statistical analysis, source power estimates were transformed into *z*-values for both conditions per subject. Permutation t-tests (independent t-tests for the GROUP effect, dependent t-test for the CONDITION effect) as implemented in fieldtrip were used to determine the brain sources of the sensor level effects. To avoid double dipping (Kriegeskorte et al. 2009), see also (Gross et al. 2013) the t-values displayed at the source plots were uncorrected for multiple comparisons and no p-values but only the peaks of the statistical maps are reported here.

#### Correlation Analysis with Beta Power

Post-hoc power in the beta frequency range (12–30 Hz) at the channels showing a significant GROUP effect in the stimulus interval and in the response interval, respectively, was subjected to a correlation analysis. Mean beta power for each subject was correlated with the subjects’ age and psychometric characteristics (IQ, AQ, EQ), as well as behavioral parameters (reaction times, SD, and correct/error rates) using Pearson correlations and we used a Holm-Bonferroni correction to correct for multiple comparisons (Gaetano 2013; Holm 1979). We set the alpha level for all significance tests at p = 0.05.

Additionally, following Mazaheri et al. (2009), we investigated single-trial correlations of power changes in different frequency bands. To this end, we chose the two sensors showing the strongest group difference in beta power for the (pre-) stimulus interval. Then, for each subject trial-by-trial pre-stimulus beta power at these channels was correlated with trial-by-trial (post) response theta, alpha, and beta power across all other sensors, resulting in topographies of frequency-specific correlations for Hits as well as FA. Time and frequency ranges for these correlations were based on the significant clusters of the sensor level analysis on time–frequency representations. In the next step, a 1-within-1-between permutation ANOVA was calculated across subjects to determine potential main effects of GROUP (NTC vs ASD) or CONDITION (Hits vs FA) or interaction effects on the correlation values. A cluster-based correction method (Maris and Oostenveld 2007b) was used to account for multiple comparisons across sensors. Last, for the significant clusters of the ANOVA the statistical significance of the correlations was also tested using one-sample t*-*tests.

## Results

### Behavioral Data

#### Number of Errors/Correct Responses

Results of statistical analyses of the behavioral parameters are shown in Table 2. As the paradigm included more Go-trials than NoGo-trials, the rate for Hits was significantly higher than the rate for FA committed in both groups (main effect of CONDITION p = 0.0002). No significant difference was found between groups; ASD and NTC committed an equal amount of errors (p = 0.26). No significant interaction was found (p = 0.14).

**Table 2:** Behavioral results and statistical analysis

Behavioral results	NTC	ASD
RTs for hits	351 ms ± 61	376 ms ± 57
RTS for false alarms	303 ms ± 34	338 ms ± 54
SD for RTs of hits	81.1 ms ± 31	153.4 ms ± 75
SD for RTs of false alarms	77.1 ms ± 54	179.2 ms ± 104
Hitrate	0.98 ± 0.02	0.96 ± 0.02
False alarm rate	0.42 ± 0.18	0.52 ± 0.22

ANOVA results	Factor	p
Reaction time	Main effect of condition	< 0.001*
	Main effect of group	0.16
	Condition X group	0.75
SD of reaction times	Main effect of condition	0.36
	Main effect of group	0.0032*
	Condition X group	0.21
Hit-/false alarm rates	Main effect of condition	< 0.001*
	Main effect of group	0.26
	Condition X group	0.14

*Bold values indicate statistical significance (p < 0.05)

#### Reaction Time and Its Variability

A main effect of CONDITION for the RT was significant (p = 0.0002). Post-hoc tests revealed that RTs for trials with FA were significantly shorter than for Hits for NTC (p = 0.0002) as well as ASD (p = 0.0052). ASD and NTC did not display any significant differences with respect to RT (p = 0.16). No significant interaction was found (p = 0.75). Concerning the variability of RT, ANOVA demonstrated a significant main effects GROUP (p = 0.0032) and an interaction GROUP and CONDITION (p = 0.044). The post-hoc tests showed a significantly higher SD of RT for Hits (p = 0.004) as well as for FA (p = 0.0088) in the ASD groups than in the NTC group. No difference between Hits and FA in SD of RT was found in NTC subjects (p = 0.47), whereas ASD patients demonstrated a tendency towards a higher SD for FA (p = 0.06).

### Neural Responses (MEG)

#### Pre-stimulus Time-Interval (CW, FA, and Response Inhibition)

**Fig. 2:**
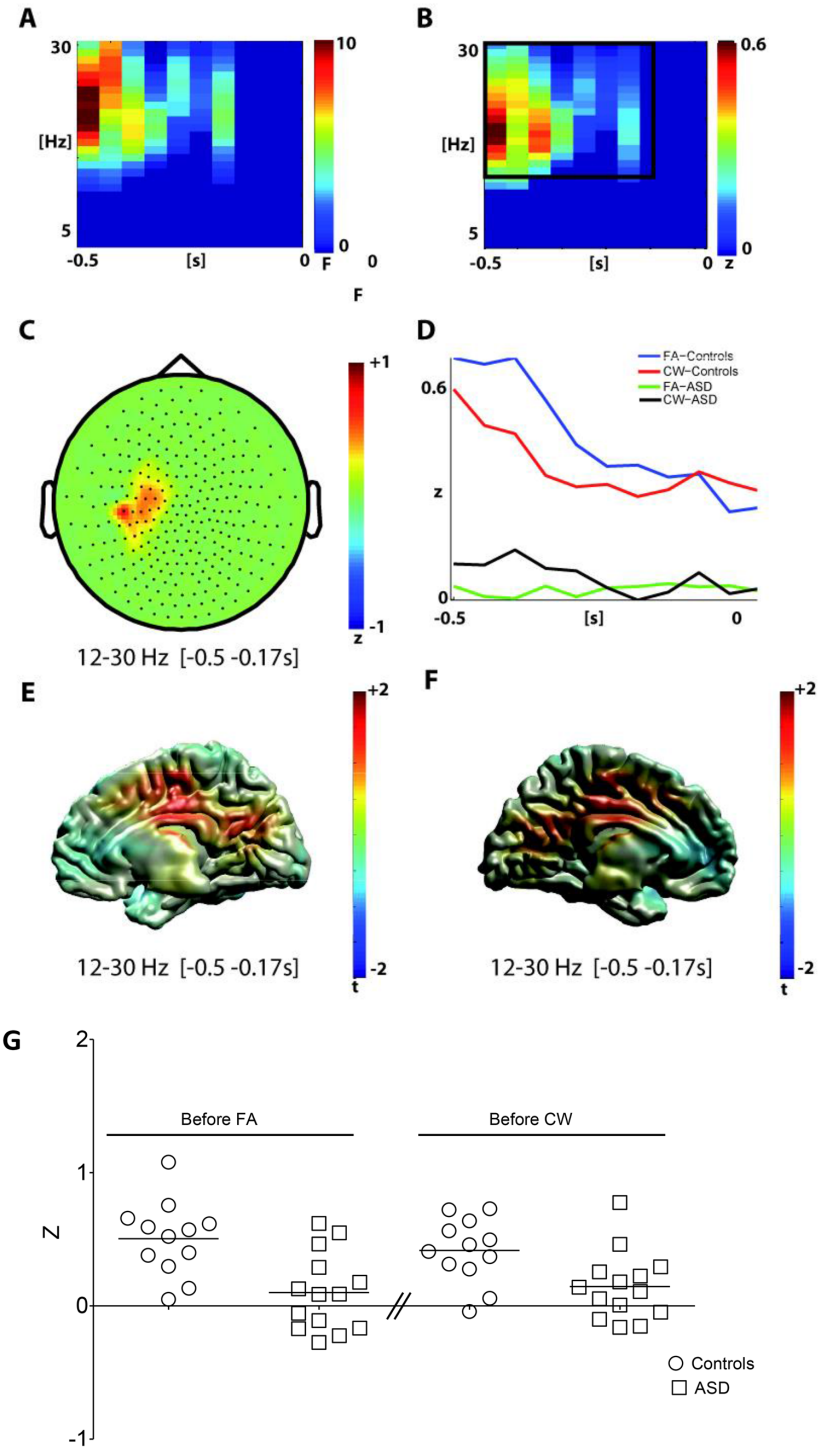
Analysis of oscillatory power in the pre-stimulus interval (− 0.5 to 0 s)—main effect of group **a** time–frequency representation of the main effect of group (controls vs ASD, 1-within 1-between permutation ANOVA, n = 26, F-values masked by p < 0.05, cluster correction, mean over significant channels shown). **b** Difference time–frequency representation in the significant cluster (controls minus ASD). The black box marks the analysis window. **c** Difference topography (controls minus ASD) in the significant cluster and analysis window. **d** Time-course of mean beta power (12–30 Hz) in the significant channels for both groups and conditions. **e**, **f** Beamformer reconstructed source power of the group effect in the pre-stimulus interval for the right (e) and left (f) hemisphere (time range − 0.5 to − 0.175 s; frequency range 12–30 Hz). g Individual pre-stimulus beta power for the time range − 0.5 to − 0.175 s; the stimulus appears at t = 0 s. *FA* false alarms, *CW* correct withholds

A significant main effect GROUP (p = 0.027; Fig. 2) was observed in a cluster of centro-parietal channels in the beta frequency band (12–30 Hz; Fig. 2a–c) approximately − 0.5 and − 0.17 s before stimulus onset. In this cluster, beta activity was significantly decreased in the ASD group for CW as well as FA (Fig. 2d). Source reconstruction localized this GROUP effect in the cingulate gyrus (peak MNI x = 0, y = -20; z = 50, Fig. 2e, f). No significant difference between CW and FA (p = 0.49) and no interaction (p = 0.41) was observed in the pre-stimulus interval.

#### Post-response Time-Interval (Hits, FA, and Post-error Adaptation)

**Fig. 3:**
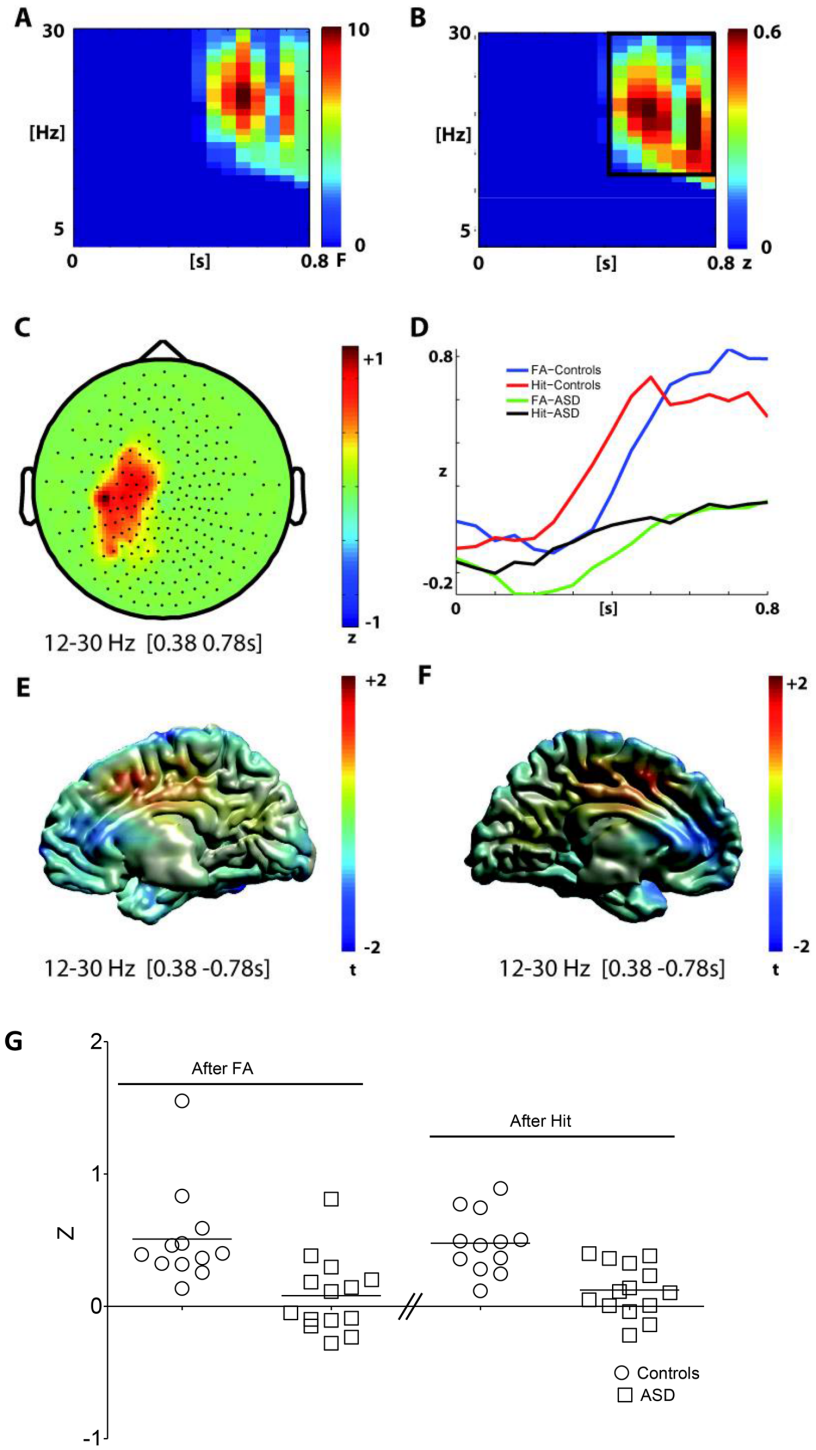
Analysis of oscillatory power in the post- response interval (0 s to 0.8 s)-Main effect of group. **a** Time–frequency representation of the main effect of group (controls vs ASD, 1-within 1-between permutation ANOVA, n = 26, F-values masked by p < 0.05, cluster correction, mean over significant channels shown). **b** Difference time–frequency representation in the significant cluster (controls minus ASD). The black box marks the analysis window. **c** Difference topography (controls minus ASD) in the significant cluster and analysis window. **d** Time-course of mean beta power (12–30 Hz) in the significant channels for both groups and conditions. **e**, **f** Beamformer reconstructed source power of the group effect in the post-response interval for the right (e) and left (f) hemisphere (time range 0.38 s to 0.78 s; frequency range 12–30 Hz). g Individual post-response beta power for the time range 0.38 s to 0.78 s. The button is pressed at t = 0 s. *FA* false alarms

The main effect GROUP was significant for the post-response interval (p = 0.012, see Fig. 3). Again, the significant cluster was located over the centro-parietal sensors (slightly more on the left side) and covered the beta frequency range (Fig. 3a–c). In the post-response interval, the time-range of the GROUP effect was approximately between 0.38 and 0.78 s after button-press. In this interval, patients with ASD showed decreased beta power in comparison with NTC subjects (Fig. 3d).

While the number of participants was relatively small, the effects were rather consistent between individuals. For individual data see Figs. 2g and 3g.

Please note that due to the mean inter-stimulus interval of 1.5 s and a response latency of around 0.34 s, the post-response interval partly overlaps with the pre-stimulus interval (see Fig. 1b).

**Fig. 4:**
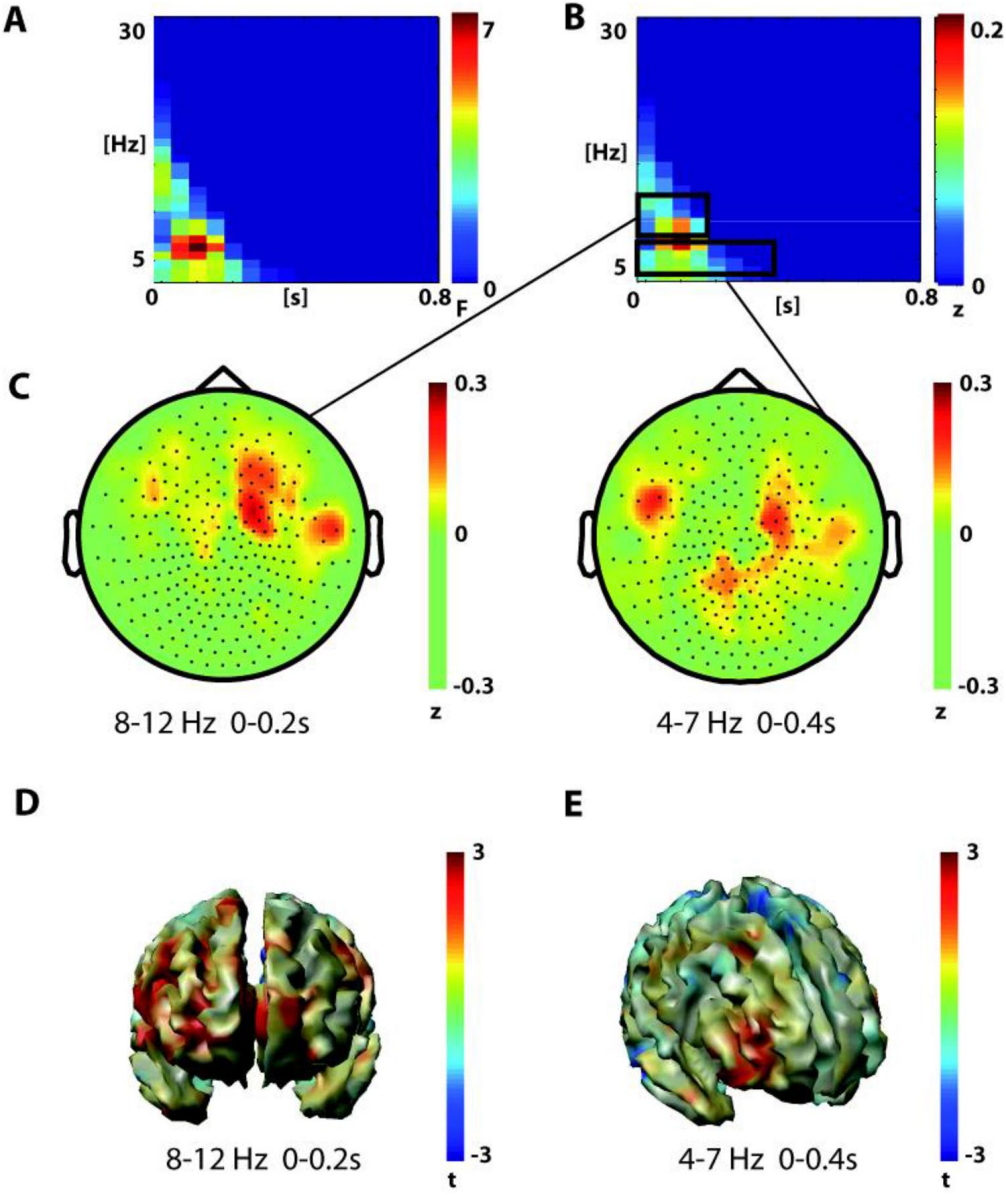
Analysis of oscillatory power in the post- response interval (0 s to 0.8 s)-Main effect of Condition—anterior cluster **a** time–frequency representation of the anterior cluster in the main effect of condition (false alarms vs hits, 1-within 1-between permutation ANOVA, n = 26, F-values masked by p < 0.05, cluster correction, mean over significant channels shown). **b** Difference time–frequency representation for the significant cluster (false alarms minus hits). Black boxes mark two analysis windows. **c**, **d** Difference topographies (false alarms minus hits) in the significant cluster and both analysis windows marked in **b**. **d**, **e** Beamformer reconstructed source power of the two analysis windows of the anterior cluster of the main effect of condition in the post-response interval. The button is pressed at = 0 s

In addition to the main effect of GROUP, in the post-response interval the main effect of CONDITION was also significant in two clusters. One cluster covered an early time range up to 0.4 s after response onset and a frequency range from 4 to 12 Hz (p = 0.012 Fig. 4). Both theta (4–7 Hz) and alpha power (8–12 Hz) were higher after FA in comparison to Hits in this early time range—with the theta increase being localized to central and bilateral fronto-temporal sensors, while the alpha increase was mostly localized to right fronto-temporal sensors (Fig. 4c). Source analysis revealed peaks of the alpha increase originating in frontal pole (MNI: x = 20; y = 40; y = 10) and the theta increase elicited in right middle frontal gyrus/orbitofrontal cortex (MNI: x = 40; y = 40; z = –10).

**Fig. 5:**
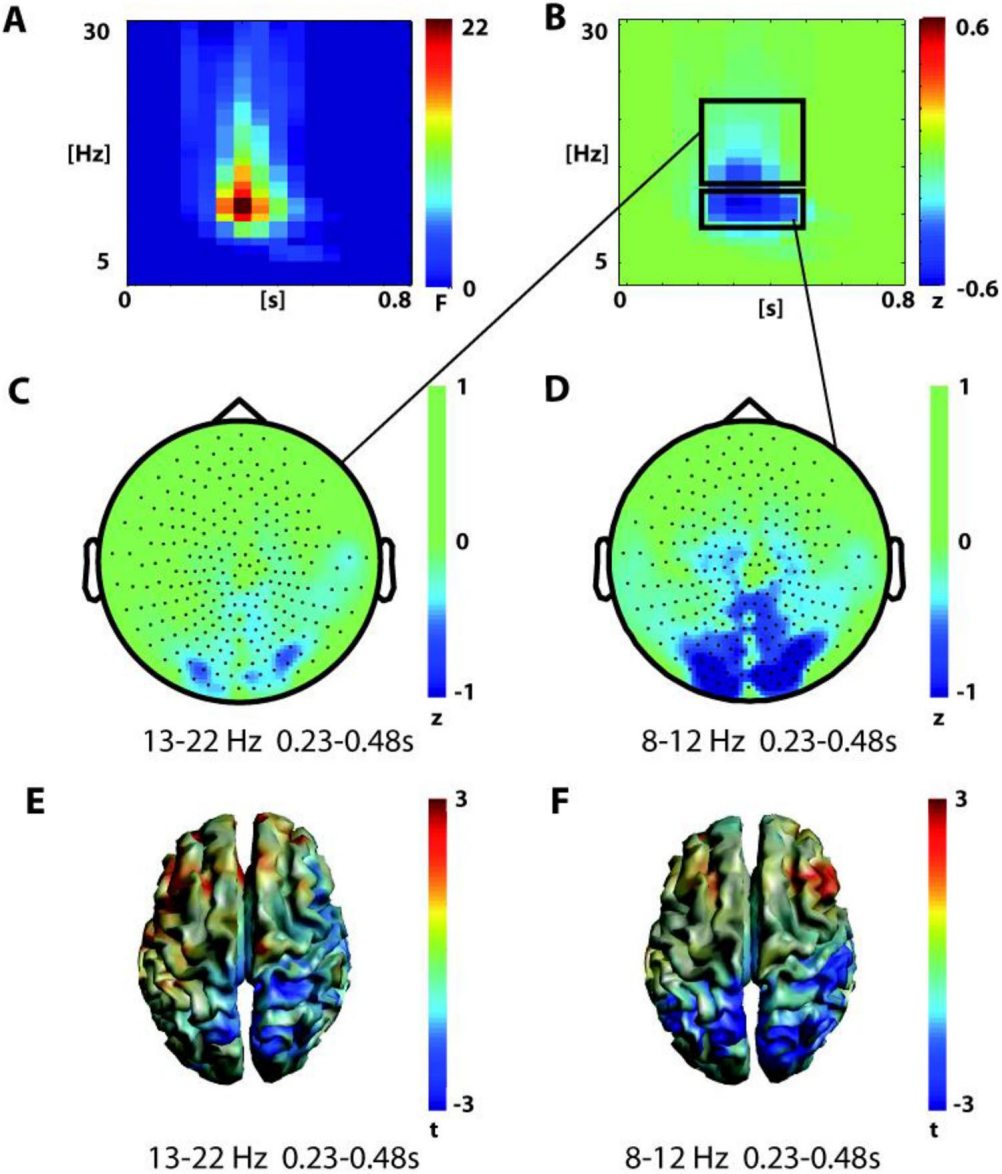
Analysis of oscillatory power in the post-response interval (0 s to 0.8 s)—main effect of condition—posterior cluster **a** time–frequency representation of the posterior cluster in the main effect of condition (false alarms vs hits, 1-within 1-between permutation ANOVA, n = 26, F-values masked by p < 0.05, cluster correction, mean over significant channels shown. **b** Difference time–frequency representation in the significant cluster (false alarms minus hits). The black boxes mark the two analysis windows. **c**, **d** Difference topographies (false alarms minus hits) in the significant cluster and both analysis windows marked in **b**. **d**, **e** Beamformer reconstructed source power of the two analysis windows of the posterior cluster of the condition effect in the post- response interval. The button is pressed at = 0 s

The other significant cluster of the CONDITION effect in the post-response interval covered a later time interval from about 0.38 to 0.78 s after button press and a frequency range from 8 to 22 Hz (p = 0.001, Fig. 5). Both alpha (8–12 Hz) and beta (13–22 Hz) power were lower after FA in comparison to Hits in this later time interval (Fig. 5b). The alpha decrease was most pronounced over central and posterior channels and the beta decrease over posterior channels (Fig. 5c). Source analysis localized the effect of alpha decrease in the occipital cortex (peak MNI x = 40; y = -70; z = 10) and the effect of beta decrease in the occipital cortex and superior parietal lobule (peak MNI x = 20, y = -70; z = 70) (Fig. 5d, e). In sum, FA first led to an increase in temporo-frontal theta to alpha activation and subsequently to a decrease in alpha to beta activity at central and posterior sensors. No significant interaction effect (p = 0.3) was found for the post-response interval.

#### Correlation of Beta Power with Psychometric Characteristics and Task Performance

To investigate whether the observed GROUP difference in beta power in the pre-stimulus and post-response interval are indicative of task performance or disease severity, we calculated post-hoc correlations between mean beta power in the significant clusters across subjects with the subjects’ characteristics and behavioral parameters (Table 3).

**Table 3:** Correlation of beta power with subjects characteristics and task performance

Pearson correlation	IQ	Age	EQ	AQ	Reaction times	SD of reaction times	Hit-/false alarm rates
Pre-stimulus; Beta power before false alarms	r = 0.14; p > 0.05	r = 0.33; p > 0.05	r = 0.42; p > 0.05	r = − 0.38; p > 0.05	r = − 0.09; p > 0.05	r = − 0.26; p > 0.05	r = − 0.06; p > 0.05
Pre-stimulus; Beta power before correct withholds	r = 0.03; p > 0.05	r = 0.52; p = 0.018*	r = 0.38; p > 0.05	r = − 0.25; p > 0.05	r = 0.04; p > 0.05	r = 0.01; p > 0.05	r = − 0.01; p > 0.05
Post-response; Beta power after false alarms	r = 0.11; p > 0.05	r = 0.42; p > 0.05	r = 0.35; p > 0.05	r = − 0.28; p > 0.05	r = − 0.15; p > 0.05	r = − 0.39; p > 0.05	r = − 0.06; p > 0.05
Post-response; Beta power after hits	r = 0.08; p > 0.05	r = 0.35; p > 0.05	r = 0.44; p = 0.04*	r = − 0.46; p = 0.04*	r = − 0.03; p > 0.05	r = − 0.2; p > 0.05	r = 0.08; p > 0.05

p values were corrected for multiple comparisons using the Holm–Bonferroni method (Gaetano 2013; Holm 1979)

*EQ* empathy quotient (Baron-Cohen and Wheelwright 2004), *AQ* autism spectrum quotient (Baron-Cohen et al. 2001)

*Bold values indicate statistical significance (p < 0.05)

We found a positive correlation between post-response beta-band power and the EQ measures of autistic traits (p = 0.04); however, there was a negative correlation between the same beta-band power and AQ (p = 0.04). Apart from these correlations of interest, the control variable ‘age of subjects’ was positively correlated with pre-stimulus beta power before CW (p = 0.018).

Due to these correlations of beta power and age, we conducted a control analysis. Both groups were matched for age (4 of the oldest controls and 5 of the youngest patients were removed, t-test on age p = 0.5) in order to check whether the GROUP effects on beta power were only caused by age differences between groups. Recalculation of the GROUP effects with 1-within-1-between ANOVA on time–frequency representations confirmed GROUP effect (p = 0.027) in the pre-stimulus interval with the same sign, location, time, and frequency range. In the post-response interval, the GROUP effect did not reach significance (p = 0.07) with the smaller sample size but showed the same sign, location, time, and frequency range (data not shown). These findings indicate that the effects observed with the complete sample were not due to group differences in age (Table 4).

**Table 4:** Correlation analysis matched for age (8 controls, 9 patients, 4 oldest controls and 5 youngest patients removed, t-test for age p = 0.5)

Pearson correlation	IQ	Age	EQ	AQ
Pre-stimulus; Beta power before false alarms	r = 0.15; p > 0.05	r = 0.3; p > 0.05	r = 0.58; p = 0.04*	r = − 0.48; p > 0.05﻿
Pre-stimulus; Beta power before correct withholds	r = 0.01; p > 0.05	r = 0.59; p = 0.03*	r = 0.45; p > 0.05	r = − 0.31; p > 0.05
Post-response; Beta power after false alarms	r = 0.16; p > 0.05	r = 0.39; p > 0.05	r = 0.47; p > 0.05	r = − 0.28; p > 0.05
Post-response; Beta power after hits	r = − 0.03; p > 0.05	r = 0.24; p > 0.05	r = 0.68; p = 0.008*	r = − 0.535; p = 0.04*

p values were corrected for multiple comparisons using the Holm-Bonferroni method (Gaetano 2013; Holm 1979)

*EQ* empathy quotient (Baron-Cohen and Wheelwright 2004), *AQ* autism spectrum quotient (Baron-Cohen et al. 2001)

*Bold values indicate statistical significance (p < 0.05)

#### Single-Trial Correlations of Pre-stimulus Beta Power with Post-response Theta, Alpha or Beta Power

**Fig. 6:**
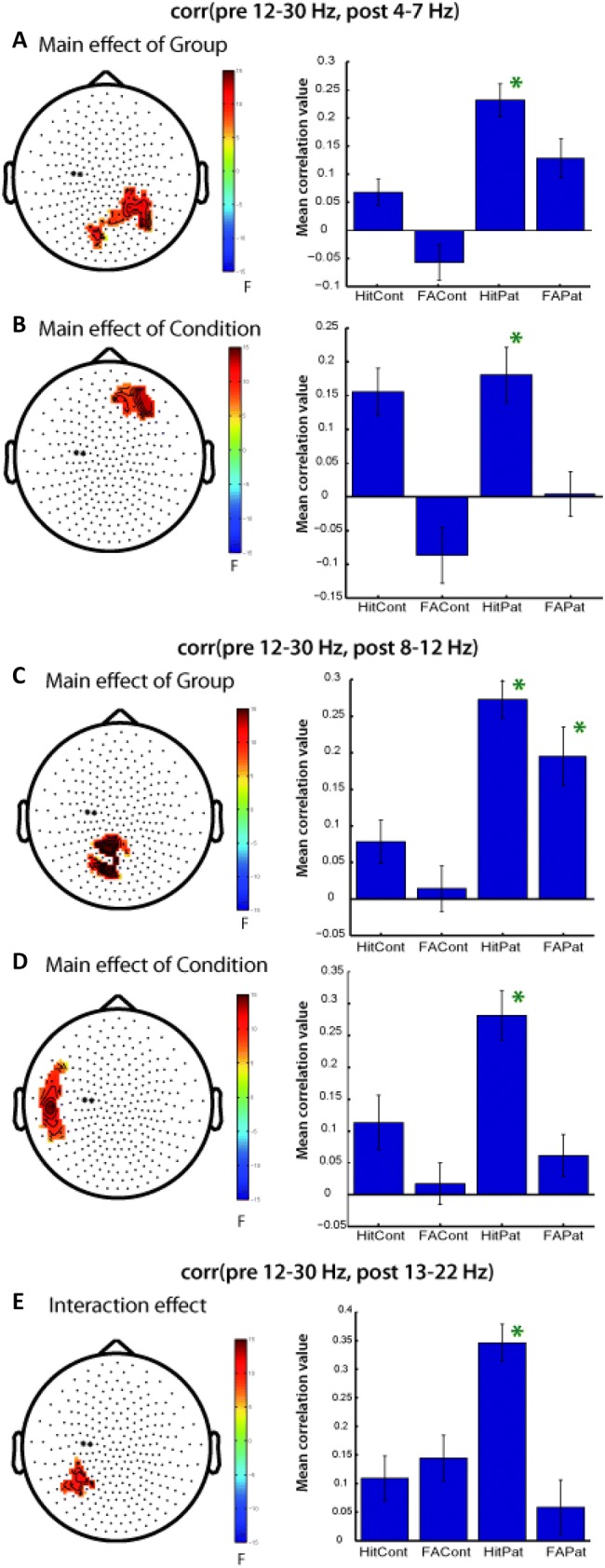
Correlation between pre-stimulus beta and post response power. Beta (12–30 Hz) power in the pre-stimulus interval (− 0.5 s to stimulus onset) of two centro-parietal sensors (marked with black asterisks on the topographies) was correlated with post response power (0–0.5 s after response) in all other sensors on a trial by trial basis. Left: topographies of results of 1-within-1-between permutation ANOVA on correlation values, n = 26, only significant clusters are depicted). Right: mean correlation values over significant channels. Green asterisks indicate also a significant one-sample t-test over the channels in the significant cluster. *HitCont* hits controls, *HitPat* hits patients, *FACont* false alarms Controls, *FAPat* false alarms patients; **a**, **b** Correlation of pre-stimulus beta power with post-response theta power (4–7 Hz). **c**, **d** Correlation of pre-stimulus beta power with post-response alpha power (8–12 Hz). **e** Correlation of pre-stimulus beta power with post-response beta power (13–22 Hz)

Based on the method proposed by Mazaheri et al. (2009), we further investigated single-trial correlations in power changes in different frequency bands. Such correlations would hint at a predictive value of pre-stimulus beta for subsequent oscillations as well as performance. Individual trial-by-trial pre-stimulus beta power was correlated with trial-by-trial post-response theta, alpha, and beta power across all other sensors. 1-within-1-between permutation ANOVAs were calculated on the obtained individual correlation values with the factors GROUP (NTC vs ASD) and CONDITION (Hits and FA). These revealed a significant main effect of GROUP (p = 0.019 and p = 0.02) over posterior channels (Fig. 6a, c, left) for the correlation of pre-stimulus beta (12–30 Hz) with post-response theta (4–7 Hz) as well as with post-response alpha (8–12 Hz). For both main effects of GROUP in the posterior clusters we found patients with ASD to show a stronger positive correlation than controls (Fig. 6a, c, right).

The ANOVA on correlation values of pre-stimulus beta with post-response theta and alpha also showed a main effect of CONDITION (p = 0.036 and p = 0.04) over right frontal channels for theta (Fig. 6b, left) and left fronto-temporal channels for alpha (Fig. 6d, left). For both CONDITION effects, we found the positive correlation to be stronger for Hits in comparison to FA (Fig. 6b, d, right). Last, for the correlation of pre-stimulus beta with post-response beta (13–22 Hz) we observed an interaction over left posterior channels (p = 0.046; Fig. 6e, left). Here, positive correlations were stronger for Hits than FA in patients, whereas the pattern was reversed in controls (i.e., stronger correlation for FA than Hits; Fig. 6e, right). Post-hoc one-sample t-tests revealed that for all significant ANOVA effects the correlations for Hits in patients with ASD were significantly different from zero. In addition, for the GROUP effect in the correlation of pre-stimulus beta and post-response alpha, also the correlation for FA in patients reached significance (Fig. 6c, right).

## Discussion

### Summary of Findings

The goal of the present study was to examine the neural processes underlying response inhibition, post-error adaptation, and response variability in patients with ASD. We focused on oscillatory changes in the MEG signals during a Go/NoGo task. The main findings are as follows:

1. At the behavioral level, subjects with ASD showed greater reaction time variability than NTC, whereas both groups were comparable with respect to overall error rates and RTs as well as the CONDITION effect (faster during FA than Hits in both groups). Therefore, performance instability was generally found in patients with ASD but it was not related to abnormal performance measures given a lack of differences between groups HITS and FA.
2. MEG analysis revealed the temporal dynamics of response inhibition (CW; FA) and post-error adaptation (Hits; FA): GROUP effects were evident for both cognitive processes.
  In the beta band (12–30 Hz), the ASD group showed decreased power in comparison to the NTC group during response inhibition over centro-parietal channels as well as after errors over the cingulate gyrus. A lack of main effect or interaction involving the factor CONDITION suggests that error-related processing (FA vs HITS; FA vs CW) was comparable between groups in the beta band.
  In the other analyzed frequency bands (theta; alpha), there were no significant GROUP differences in the pre-stimulus phase.
3. By contrast, there were error-related effects on theta and alpha in the post-response interval: The dynamic of post-response alpha after errors (FA) compared with correct responses (Hits) was characterized by an initial enhancement (~ 0 s to 0.4 s) at fronto-temporal sensors and concomitant with a theta increase. Subsequently, there was a decrease (~ 0.4 s – 0.8 s) over central and posterior sensors concomitant with lower beta power. This pattern was present irrespective of group membership (no GROUP or interaction effect).
4. Correlation analysis on mean beta power in these GROUP specific clusters revealed an association between autism-related parameters like EQ (positive correlation) and AQ (negative correlation). This result was independent of CONDITION (Hits; FA) and thus not error-specific. Additionally, RT within-subject variability on FA trials negatively correlated with post-response beta levels in both groups.
5. We found single trial correlations of pre-stimulus beta power with post-response theta and alpha power that were stronger for ASD than NTC and for HITS than FA. With respect to post-response beta power, an antagonistic pattern showed that intensity of pre-stimulus beta before HITS predicted enhanced beta values in ASD compared to FA, whereas the control group demonstrated more beta power after FA than HITS.

#### (1) Comparable Performance But Enhanced Intra-Subject Variability in ASD

First, as expected RTs for FA were significantly shorter than for Hits in both groups, replicating previous findings of shorter RTs for error trials compared to correct responses (Manly et al. 1999; Mazaheri et al. 2009; Robertson et al. 1997). In agreement with Robertson and colleagues (Robertson et al. 1997), this provides evidence for a temporary lapse in attention during which participants respond automatically without top-down control over signals, leading to the occurrence of prepotent responses (FA).

Second, behavioral performance in the ASD group during the Go-NoGo task revealed no impairment, as RTs and rates of button presses during Go- and NoGo-trials (both Hits and FA) were comparable between groups. These results adhere to previous reports of unaltered RTs and error rates (FA) for ASD patients (see (Ozonoff and Jensen 1999; Ozonoff and Strayer 1997; Ozonoff et al. 1994). We therefore cautiously conclude that behavioral impairment in Go-NoGo tasks cannot differentiate patients with ASD and heathy subjects.

Third, trial-by-trial RTs were more variable in ASD. Previous research on RT variability in ASD has yielded ambiguous results: In fact, enhanced intra-subject SD may be accounted for by comorbid Attention Deficit Hyperactivity Disorder (ADHD) symptoms in ASD participants (see Adamo et al. 2014; Carter Leno et al. 2018; Salunkhe et al. 2018). Still, (Karalunas et al. 2018) found comparably enhanced variability in ASD patients with/without ADHD in a continuous performance test. As attention problems were not considered in the present samples, the observed enhanced performance instability may indeed have resulted from comorbid attention deficits rather than being autism-specific. This issue should be focused on in future studies.

In sum, psychophysiological measures hint at arousal instabilities in the ASD group that did not affect overall performance (RT; error rates). The MEG parameters as well as correlations thereof shed further light on the neuronal mechanism underlying these effects and potential group differences, as outlined in the following.

#### (2) Lower Beta-Power as Putative Top-Down Control Deficit in ASD

ASD patients demonstrated lower power in the beta band during response inhibition irrespective of outcome, i.e. before CW as well as FA. As FA may reflect temporary lapses in attention (Robertson et al. 1997), we speculate that pre-stimulus beta is not associated with top-down attentional processing (no CONDITION effect). No other pre-stimulus frequency was sensitive to GROUP or CONDITION.

Moreover, we wish to point out that while suppressed pre-stimulus beta may be a sensitive marker for presence/absence of ASD, it does not appear to be indicative of subsequent performance (no interaction effect). Past studies investigating pre-stimulus oscillations in healthy individuals primarily report suppressed alpha rather than beta activity before cognitive involvement (see Bauer et al. 2014, for a discussion) either due to automatic bottom-up suppression (Bauer et al. 2006; Pfurtscheller and Lopes da Silva 1999) or by active top-down inhibitory signals (Jensen and Mazaheri 2010; Klimesch et al. 2007). Pre-stimulus beta activity on the other hand is associated with enhanced alertness and tonic (i.e. between-trial) top-down activation states (Richter et al. 2017). Accordingly, it seems that lack of tonic top-down control mechanisms may have yielded the reported group differences. Our ASD sample also showed reduced predictability values of resting state power in posterior regions of the default mode network, as reported in Brodski-Guerniero et al. (2018). In that analysis, information obtained at one time point was found to be predictive for subsequent time points and was associated with alpha and beta power (source space). However, this predictive information was reduced in the ASD group. Following (Brodski-Guerniero et al. 2018), there may be a bias in ASD towards bottom-up information due to less reliable preparatory top-down signals that are required for context updating. In addition, signal predictability arising in posterior brain regions negatively correlated with autistic symptom severity across groups in that study, hinting at a direct relation between top-down predictions and ASD abnormality. Complementarily, the present analysis on Go/NoGo performance using the same participants shows that also during task, incoming signals were apparently less efficiently processed, as evidenced by the lower pre-stimulus beta strength in the ASD sample. We speculate that this led to a non-optimal state of focused attention that is required for trial-by-trial decisions about responses and inhibitions. As performance was comparable between groups, additional phasic (within-trial) compensatory mechanisms probably took place, which will be presented in the following (see (3) and (4) below).

Apart from response inhibition (Hits; FA), post-error adaptation after FA compared to successful inhibition (CW) likewise delivered a GROUP effect of beta activity modulation in either group (no CONDITION or interaction effects). Like the lower pre-stimulus beta power in ASD, decreased post-response beta therefore appears to be generally prominent in ASD without representing a specific underlying cognitive process associated with errors. Interestingly, both beta GROUP effects (pre-stimulus and post-response) were generated in the same source regions (paracingulate/cingulate gyrus). It is therefore likely that both GROUP effects represent comparable, maybe interdependent cognitive processes. The sources are integral to the ventral attentional system (Vossel et al. 2014) associated with bottom-up processing. This finding fits with the above-mentioned suggestion of inefficient alertness in ASD compared to NTC subjects during tasks: Instead of recruiting dorsal fronto-parietal structures to achieve a proactive state, ASD patients rather seemed to react passively to task requirements. Gamma oscillations are most prominent during bottom-up reactive processing and are associated with the ventral attention system (Bastos et al. 2012) but were not focussed on here. Following this idea, future research focusing on high frequency bands in ASD during error processing can complement these findings. With this, different hypotheses on aberrant long- and short-range connections for the low and high frequency bands in ASD would be put to a test.

Finally, while beta activity prior to stimuli hints at cognitive mode differences between ASD patients and NTC subjects, pre-stimulus theta and alpha power were not deviant in ASD, nor were there any frequency differences during subsequent attentional lapses (FA) compared with correct responses (Hits). Still, correlation analysis reveals putative mechanisms that may have allowed performance comparability (see (3) and (4) below).

#### (3) Alpha Dynamics During Inhibition and Post-error Adaptation are Preserved in ASD

Commission of errors (FA) elicited a sequence of initial alpha activation and subsequent alpha suppression in both groups. A simultaneous enhancement phase was also observable for theta. In previous studies, theta increases accompanied alpha suppression and correlated with behavioral measures of post-error adaptation (e.g. Novikov et al. 2015). In our study, alpha suppression only occurred later and in combination with the reported post-response beta suppression. Alpha suppression is discussed as a correlate of selective attention (Klimesch et al. 2007) by enabling the shift into an active state that is controlled in posterior sensory regions (Clayton et al. 2015). Intriguingly, no subject from our sample showed this alpha pattern. Similarly, our results contradict findings of Mazaheri and colleagues using an analogous task (Mazaheri et al. 2009). There, higher pre-stimulus alpha-activation (10–11 Hz) preceded FA, fostering the idea of pre-stimulus alpha suppression as a proxy for level of focused attention. In the present study, we observed no such CONDITION effects for either group. One possible reason may be rooted in our sample characteristics: While Mazaheri et al. (2009) tested only healthy adults, our sample included adolescents as well. Indeed, several fMRI-studies using Go-NoGo or Stop-Signal tasks (e.g. Rubia et al. 2003; Tamm et al. 2002) have shown differences in neural activation between adolescents and adults during response inhibition. Rubia et al. (2003), for instance, compared the neural activation of adolescents (mean age = 15.01 ± 2.3 years) and adults (mean age = 28.8 ± 6.64 years) during a Go- NoGo task and found significantly greater activation for adults than adolescents in the left middle and inferior frontal gyri. Age differences between our participants (19.49 ± 3.36 years) and Mazaheri et al.’s (2009) sample (mean age of 27 years), may hence explain conflicting results patterns with respect to the pre-stimulus interval. However, this possibility is debatable because individuals in the NTC group were significantly older than those in our ASD group. Thus, based on age the same pattern of pre-stimulus activation obtained by Mazaheri et al. (2009) should have been present in the NTC group. Given comparable pre-stimulus alpha power before FA for the NTC group and the fact that neural activation between 17.8 ± 2.09 years (ASD-group) and 20.3 ± 3.7 years (NTC group) seems to be equivalent, we believe that age differences did not affect our results. This in turn suggests that the FA-associated alpha enhancement before errors reported by Mazaheri et al. (2009) may be associated with divergent sample characteristics between the studies.

Irrespective of the nature of the post-response alpha dynamic, we conclude that post-error adaptation alone was not sensitive to dissociate ASD from NTC. It seems that more sophisticated analyses of post-error adaptation are required. Thus, the correlation between pre- and post-beta activity that we found to show antagonistic processes between groups is associated with post-error adaptation (see (4) and may be a better marker for autism.

#### (4) Post-Error Adaptation in ASD as Potential Bottom-Up Compensatory Mechanism

Beta power prior to stimuli was predictive of subsequent oscillations (post-response theta, alpha, and beta) and sensitive to response outcome (HIT, FA), with enhanced theta and alpha values for HITS relative to FA. However, our results should be interpreted with caution concerning the alpha and theta frequency bands, since both bands were not distinguishable between the two groups.

Pre-stimulus activity on FA trials can be considered a marker for a non-optimal attentional state leading to errors (Robertson et al. 1997). According to the activation–suppression hypothesis (Ridderinkhof 2002), post-error adaptive behavior is characterized by slower RTs and more focused attention to meet task requirements and maintain the current task goal. These behavioral adjustments also manifest themselves in neurophysiological signatures: For example, Marco-Pallarés et al. (2008) found enhanced beta activity during inhibitory stages of post-response processing. Structures that are discussed in this context are right IFG (Swann et al. 2009) and pre-supplementary motor area (Neubert et al. 2010), which fosters the idea of beta activity being related to a motor inhibition network (Aron et al. 2007).

Interestingly, the correlation analysis further dissociated ASD from NTC based on antagonistic relations between pre-stimulus beta and post-response beta activity. The correlation strength was higher for HITS than FA in ASD patients but higher for FA than HITS in NTC. It seems that beta activity is more involved in ASD to maintain task goals (HITS) but primarily associated with attentional lapses (FA) in NTC subjects. This may stem from phasic (within-trial) compensatory mechanisms keeping ASD patients on track during the Go/NoGo task as opposed to tonic (between-trial) adaptation processes in the control group. These findings are thus consistent with the aforementioned evidence for reactive (phasic) processing in ASD patients during task. On a broader scale, this provides possible evidence for the complex interplay between inhibition, post-error adaptation, and response variability and may contribute to reconciling contradictory theories on ASD.

#### (5) Beta Activity as Indicator of ASD and RT Stability

The hypothesis of a reactive processing mode in ASD is in line with the association we observed between beta-power and autistic traits. In contrast to the aforementioned results, here we detected error-specific differences between groups. On FA trials, lower pre- and post-stimulus beta activity predicted higher levels of autistic traits (EQ; AQ). Additionally, post-beta on FA trials correlated with response variability (lower beta values predicted higher SD). It should be noted that RTs were also less stable on FA trials in ASD, whereas overall RTs, error rates, and variability on Hit trials were comparable. Therefore, trial-by-trial variations of RTs potentially represent a compensatory mechanism that is neurophysiologically reflected in beta abnormalities: ASD patients possibly reacted to stimuli in a more cautious state during stimulus presentation (lower pre-stimulus beta) without establishing a stable top-down control mode between trials (lower post-response beta). Such trial-by-trial variations with lacking adaptation efforts could lead to correlations between pre-stimulus and post-response phases, i.e. highly related phasic (within-trial) oscillations. Similar deviations are apparently involved in ADHD, as Grane et al. (2016) report attenuated attentional resource allocation in response to reactive control during a Go/NoGo task in an ADHD sample. Findings on cognitive control modes in ASD are largely lacking (see Lever et al. 2016), though preliminary research corroborates the present suggestions. Thus, ASD is associated with altered functional connectivity patterns in ventral fronto-parietal regions putatively subserving bottom-up-driven attention (e.g. Larson et al. 2012; Solomon et al. 2014). However, these neuronal abnormalities did not translate into performance differences between groups. This parallels our finding of lower pre-stimulus beta activity in ASD than controls that we interpret to represent enhanced bottom-up suppression of incoming signals. Indeed, correlation analysis of pre-stimulus beta with post-response frequencies provides additional support for reactive control in ASD.

## Limitations

One limitation of this study was that comorbid symptoms of ADHD were not controlled. Due to the high prevalence of comorbid ASD and ADHD with a range of 30% (Leyfer et al. 2006; Simonoff et al. 2008) up to 90% (Witwer and Lecavalier 2010), all patients with a primary ASD diagnosis were include in the sample. Future studies should investigate whether the suggested compensatory mechanisms are due to inattentiveness grounded in ADHD or genuine ASD-related processes. Likewise, age differences may explain inhibitory control problems in ASD (e.g. Ozonoff and Jensen 1999; Ozonoff and Strayer 1997; Ozonoff et al. 1994; Ozonoff et al. 2011). In the present study, the ASD group was slightly younger than NTC, while did not differ significantly. Still, future studies should be conducted with age-matched groups.

Furthermore, no enhanced pre-stimulus beta power was observable in the NTC group, whereas beta power is frequently associated with tonic top-down enhancement before stimuli. We speculate that this is due to our analyses focusing on phasic within-trial changes rather than tonic between-trial differences. In turn, this exposes the need for future studies to address the full frequency range with respect to a putative ASD-related connectopathy.

As a general remark, cognitive control is no singular operation but is composed of different executive functions, and many paradigms have been developed to test different subcomponents. While the present results provide insights into response inhibition, post-error adjustment, and response variability in a Go/NoGo task, other mechanisms remain uncovered, e.g. conflict-related inhibition. These require further investigations in future studies.

## Conclusion

The present study addressed putative differences between ASD and NTC in neuronal processing of response inhibition, post-error adjustment, and response variability. We identified beta oscillations rather than behavioral parameters as key features reflecting dysfunctions in ASD. In particular, while ASD patients were not behaviorally impaired when considering performance over the entire experiment (mean RTs; error rates), enhanced intra-subject RT intra-subject variability apparently revealed compensatory mechanisms. These in turn may be mediated by within-trial reactive means to overcome a non-optimal state of between-trial arousal. Abnormal pre-stimulus as well as post-response beta oscillations in ASD correlated with enhanced variability irrespective of response outcome (correct; erroneous). This fosters the idea that phasic countermeasures sufficed to keep up with the healthy control group. The latter showed typical post-error adaptation oscillations in the beta range and a lower RT variability.

Lack of adjustment to changing conditions is also associated with clinical symptoms in ASD in terms of rigid, repetitive behaviors. This may well be grounded on difficulties to inhibit prepotent responses and to update the current task goal. Our study therefore provides possible neurophysiological evidence for a possible endophenotype of ASD.

In sum, we postulate that a bottom-up reactive mode was predominant in ASD and translated primarily into abnormal beta activity.
